# 5-Phenyl-3,4,4a,5,6,12c-hexa­hydro-2*H*-benzo[*f*]pyrano[3,2-*c*]quinoline

**DOI:** 10.1107/S1600536810020787

**Published:** 2010-06-09

**Authors:** Bai-Xiang Du, Jie Zhou, Yu-Ling Li, Xiang-Shan Wang

**Affiliations:** a School of Chemistry and Chemical Engineering, Xuzhou Normal University, Xuzhou Jiangsu 221116, People’s Republic of China

## Abstract

In the title compound, C_22_H_21_N, the pyridine ring adopts a distorted boat conformation, while the adjacent pyran ring adopts a chair conformation; the heterocyclic rings make a dihedral angle of 40.1 (2)° with each other.

## Related literature

For the biological properties of pyran­oquinoline derivatives, see: Faber *et al.* (1984 ▶); Johnson *et al.* (1989 ▶); Schiemann *et al.* (2007 ▶); Yamada *et al.* (1992 ▶). Zhao & Teng (2008 ▶). For related structures, see: Ramesh *et al.* (2008 ▶); Zhao & Teng (2008 ▶); Bai *et al.* (2009 ▶).
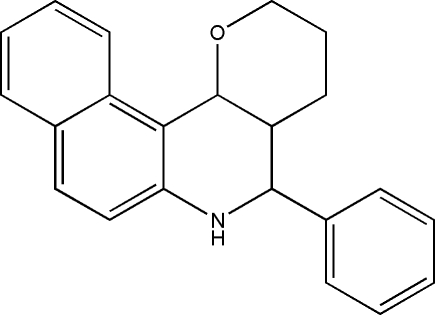


## Experimental

### 

#### Crystal data

C_22_H_21_NO
*M* *_r_* = 315.40
Monoclinic, 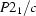

*a* = 8.1106 (2) Å
*b* = 10.9560 (2) Å
*c* = 18.5020 (3) Å
β = 93.552 (1)°
*V* = 1640.92 (6) Å^3^
*Z* = 4
Mo *K*α radiation
μ = 0.08 mm^−1^
*T* = 296 K
0.50 × 0.33 × 0.10 mm

#### Data collection

Bruker APEXII area-detector diffractometer
11135 measured reflections
2956 independent reflections
2303 reflections with *I* > 2σ(*I*)
*R*_int_ = 0.023

#### Refinement

*R*[*F* ^2^ > 2σ(*F* ^2^)] = 0.047
*wR*(*F* ^2^) = 0.140
*S* = 1.03
2956 reflections
221 parameters
1 restraint
H-atom parameters constrained
Δρ_max_ = 0.30 e Å^−3^
Δρ_min_ = −0.21 e Å^−3^

### 

Data collection: *SMART* (Bruker, 2001 ▶); cell refinement: *SAINT* (Bruker, 2001 ▶); data reduction: *SAINT*; program(s) used to solve structure: *SHELXS97* (Sheldrick, 2008 ▶); program(s) used to refine structure: *SHELXL97* (Sheldrick, 2008 ▶); molecular graphics: *SHELXTL* (Sheldrick, 2008 ▶); software used to prepare material for publication: *SHELXTL*.

## Supplementary Material

Crystal structure: contains datablocks global, I. DOI: 10.1107/S1600536810020787/pv2287sup1.cif

Structure factors: contains datablocks I. DOI: 10.1107/S1600536810020787/pv2287Isup2.hkl

Additional supplementary materials:  crystallographic information; 3D view; checkCIF report

## Figures and Tables

**Fig. 1.**
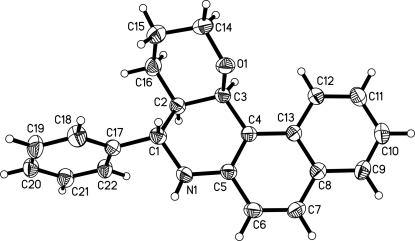
The molecular structure drawing for (I) showing 30% probability of displacement ellipsoids and the atom-numbering scheme.

**Fig. 2.**
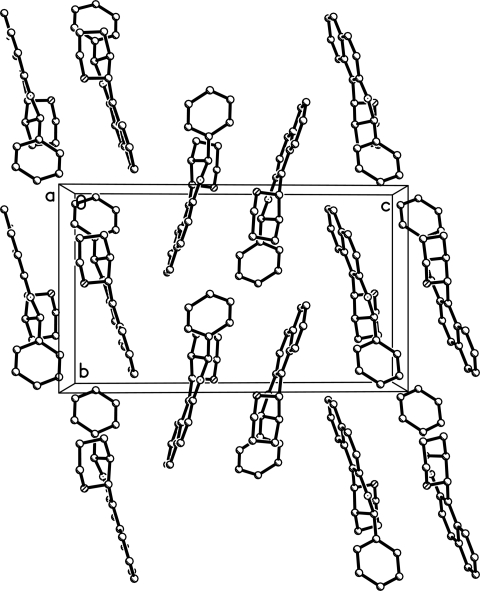
The molecular packing diagram of (I).

## supplementary crystallographic information

### Comment

The synthesis of pyranoquinoline derivatives has been the focus of great interest, because it was reported that its derivatives possessed a broad spectrum of biological properties. Some of these activities include psychotropic activity (Yamada *et al.*, 1992), anti-allergenic activity (Faber *et al.*, 1984), and anti-inflammatory (Johnson *et al.*, 1989). They are also used for the treatment of proliferative diseases, such as cancer (Schiemann *et al.*, 2007). The title compound may be used as a new precursor for obtaining bioactive molecules. We report here the crystal structure of the title compound, (I).

In the crystal structure of (I), the pyridine ring of the pyranoquinoline moiety is slightly distorted and adopts a distorted boat conformation (Fig. 1). The atoms C1 and C2 deviate from the basal plane defined by the atoms C3—C5/N1 by 0.253 (3) and -0.495 (3) Å, respectively. This conformation is similar to that found in other hydropyridine derivatives (Ramesh *et al.* 2008; Zhao & Teng, 2008; Bai *et al.*, 2009). In the adjacent pyran ring, the atoms C2, C3, C14 and C15 are coplanar, while the atoms O1 and C16 deviate from the plane by 0.659 (3) and -0.623 (3) Å, respectively. These data indicate that the pyran ring adopts a chair confirmation. The basal plane of the pyridine ring nearly parallel to the naphthalene ring C4—C13, forming a dihedral angle of 2.7 (1)°, and makes a dihedral angle of 82.2 (1)° to benzene ring. Two heterocyclic rings make a dihedral angle of 40.1 (1)°.

### Experimental

The title compound, (I), was prepared by the reaction of benzaldehyde (0.212 g, 2 mmol), naphthalen-2-amine (0.286 g, 2.0 mmol), 3,4-dihydro-2*H*-pyran (0.252 g, 3.0 mmol), I_2_ (0.026 g, 0.1 mmol) and THF (10 ml) for 14 h (yield 86%, mp. 477–478 K). Crystals of (I) suitable for X-ray diffraction were obtained by slow evaporation of a dimethylformamide (dmf) solution.

### Refinement

The H atoms were calculated geometrically and refined as riding, with C—H = 0.93–0.98 Å, except for H1 which was located from a difference map and its distance was restricted at 0.85 by DFIX command, with *U*_iso_(H) = 1.2*U*_eq_(parent atom).

### Crystal data

**Table:**

C_22_H_21_NO	*F*(000) = 672
*M**_r_* = 315.40	*D*_x_ = 1.277 Mg m^−^^3^
Monoclinic, *P*2_1_/*c*	Melting point = 477–478 K
Hall symbol: -P 2ybc	Mo *K*α radiation, λ = 0.71073 Å
*a* = 8.1106 (2) Å	Cell parameters from 3365 reflections
*b* = 10.9560 (2) Å	θ = 2.5–26.4°
*c* = 18.5020 (3) Å	µ = 0.08 mm^−^^1^
β = 93.552 (1)°	*T* = 296 K
*V* = 1640.92 (6) Å^3^	Block, colourless
*Z* = 4	0.50 × 0.33 × 0.10 mm

### Data collection

**Table:**

Bruker APEXII area-detector diffractometer	2303 reflections with *I* > 2σ(*I*)
Radiation source: fine-focus sealed tube	*R*_int_ = 0.023
graphite	θ_max_ = 25.2°, θ_min_ = 2.2°
φ & ω scans	*h* = −9→9
11135 measured reflections	*k* = −13→12
2956 independent reflections	*l* = −22→22

### Refinement

**Table:**

Refinement on *F*^2^	Primary atom site location: structure-invariant direct methods
Least-squares matrix: full	Secondary atom site location: difference Fourier map
*R*[*F*^2^ > 2σ(*F*^2^)] = 0.047	Hydrogen site location: inferred from neighbouring sites
*wR*(*F*^2^) = 0.140	H-atom parameters constrained
*S* = 1.02	*w* = 1/[σ^2^(*F*_o_^2^) + (0.0712*P*)^2^ + 0.4581*P*] where *P* = (*F*_o_^2^ + 2*F*_c_^2^)/3
2956 reflections	(Δ/σ)_max_ < 0.001
221 parameters	Δρ_max_ = 0.30 e Å^−^^3^
1 restraint	Δρ_min_ = −0.21 e Å^−^^3^

### Special details

**Table:**

Geometry. All e.s.d.'s (except the e.s.d. in the dihedral angle between two l.s. planes) are estimated using the full covariance matrix. The cell e.s.d.'s are taken into account individually in the estimation of e.s.d.'s in distances, angles and torsion angles; correlations between e.s.d.'s in cell parameters are only used when they are defined by crystal symmetry. An approximate (isotropic) treatment of cell e.s.d.'s is used for estimating e.s.d.'s involving l.s. planes.
Refinement. Refinement of *F*^2^ against ALL reflections. The weighted *R*-factor *wR* and goodness of fit *S* are based on *F*^2^, conventional *R*-factors *R* are based on *F*, with *F* set to zero for negative *F*^2^. The threshold expression of *F*^2^ > σ(*F*^2^) is used only for calculating *R*-factors(gt) *etc*. and is not relevant to the choice of reflections for refinement. *R*-factors based on *F*^2^ are statistically about twice as large as those based on *F*, and *R*- factors based on ALL data will be even larger.

### Fractional atomic coordinates and isotropic or equivalent isotropic displacement parameters (Å^2^)

**Table:**

	*x*	*y*	*z*	*U*_iso_*/*U*_eq_	
C4	0.3714 (2)	1.07478 (15)	0.36093 (9)	0.0408 (4)	
N1	0.61481 (19)	0.95714 (14)	0.39789 (10)	0.0537 (4)	
O1	0.18206 (15)	0.97459 (12)	0.43549 (7)	0.0562 (4)	
C13	0.3017 (2)	1.18875 (15)	0.33707 (9)	0.0414 (4)	
C5	0.5404 (2)	1.06466 (16)	0.37471 (10)	0.0439 (4)	
C3	0.2648 (2)	0.96399 (16)	0.36947 (10)	0.0442 (4)	
H3A	0.1808	0.9625	0.3291	0.053*	
C8	0.4069 (2)	1.29013 (16)	0.32593 (9)	0.0452 (4)	
C6	0.6433 (2)	1.16641 (18)	0.36261 (11)	0.0544 (5)	
H6A	0.7571	1.1589	0.3711	0.065*	
C1	0.5156 (2)	0.85413 (16)	0.42038 (10)	0.0470 (4)	
H1A	0.4798	0.8705	0.4691	0.056*	
C2	0.3629 (2)	0.84524 (16)	0.36796 (10)	0.0467 (4)	
H2A	0.4012	0.8359	0.3191	0.056*	
C7	0.5791 (2)	1.27409 (18)	0.33898 (11)	0.0529 (5)	
H7A	0.6497	1.3390	0.3311	0.064*	
C17	0.6191 (2)	0.73967 (16)	0.42288 (9)	0.0439 (4)	
C10	0.1721 (3)	1.41656 (19)	0.29126 (12)	0.0623 (6)	
H10A	0.1281	1.4914	0.2761	0.075*	
C22	0.6980 (2)	0.69912 (17)	0.36296 (10)	0.0522 (5)	
H22A	0.6887	0.7443	0.3203	0.063*	
C9	0.3376 (3)	1.40304 (17)	0.30283 (10)	0.0552 (5)	
H9A	0.4068	1.4690	0.2955	0.066*	
C11	0.0673 (3)	1.31761 (19)	0.30220 (12)	0.0614 (5)	
H11A	−0.0462	1.3270	0.2940	0.074*	
C12	0.1299 (2)	1.20753 (17)	0.32474 (10)	0.0515 (5)	
H12A	0.0579	1.1433	0.3322	0.062*	
C16	0.2505 (3)	0.73714 (18)	0.38181 (12)	0.0603 (5)	
H16A	0.1730	0.7260	0.3403	0.072*	
H16B	0.3168	0.6637	0.3874	0.072*	
C18	0.6379 (3)	0.6707 (2)	0.48508 (11)	0.0630 (6)	
H18A	0.5869	0.6954	0.5263	0.076*	
C21	0.7894 (3)	0.59365 (18)	0.36538 (11)	0.0581 (5)	
H21A	0.8399	0.5676	0.3243	0.070*	
C20	0.8067 (3)	0.52664 (18)	0.42770 (12)	0.0604 (5)	
H20A	0.8693	0.4555	0.4293	0.073*	
C15	0.1569 (3)	0.7550 (2)	0.44793 (14)	0.0697 (6)	
H15A	0.2325	0.7529	0.4907	0.084*	
H15B	0.0773	0.6896	0.4517	0.084*	
C14	0.0693 (3)	0.8750 (2)	0.44381 (14)	0.0692 (6)	
H14A	−0.0120	0.8742	0.4031	0.083*	
H14B	0.0115	0.8871	0.4876	0.083*	
C19	0.7316 (3)	0.5649 (2)	0.48709 (12)	0.0710 (6)	
H19A	0.7431	0.5197	0.5296	0.085*	
H1	0.7087	0.965	0.4204	0.087*	

### Atomic displacement parameters (Å^2^)

**Table:**

	*U*^11^	*U*^22^	*U*^33^	*U*^12^	*U*^13^	*U*^23^
C4	0.0409 (9)	0.0403 (9)	0.0416 (9)	−0.0009 (7)	0.0053 (7)	−0.0061 (7)
N1	0.0389 (8)	0.0454 (9)	0.0761 (11)	0.0008 (7)	−0.0021 (8)	−0.0027 (8)
O1	0.0504 (7)	0.0515 (8)	0.0685 (9)	−0.0046 (6)	0.0189 (6)	−0.0062 (6)
C13	0.0459 (9)	0.0410 (9)	0.0376 (9)	0.0006 (7)	0.0059 (7)	−0.0056 (7)
C5	0.0405 (9)	0.0423 (9)	0.0492 (10)	0.0001 (7)	0.0049 (7)	−0.0071 (8)
C3	0.0398 (9)	0.0440 (10)	0.0488 (10)	−0.0012 (7)	0.0026 (7)	−0.0022 (8)
C8	0.0532 (10)	0.0440 (10)	0.0390 (9)	−0.0010 (8)	0.0074 (8)	−0.0064 (7)
C6	0.0398 (9)	0.0557 (12)	0.0678 (13)	−0.0048 (9)	0.0050 (9)	−0.0073 (9)
C1	0.0455 (9)	0.0462 (10)	0.0496 (10)	0.0020 (8)	0.0038 (8)	−0.0062 (8)
C2	0.0476 (10)	0.0435 (10)	0.0489 (10)	0.0003 (8)	0.0019 (8)	−0.0057 (8)
C7	0.0537 (11)	0.0472 (11)	0.0589 (12)	−0.0109 (9)	0.0112 (9)	−0.0048 (9)
C17	0.0430 (9)	0.0433 (10)	0.0454 (10)	0.0001 (7)	0.0020 (7)	−0.0028 (8)
C10	0.0745 (14)	0.0462 (11)	0.0665 (13)	0.0146 (10)	0.0071 (11)	0.0003 (9)
C22	0.0618 (11)	0.0503 (11)	0.0454 (11)	0.0115 (9)	0.0096 (9)	0.0059 (8)
C9	0.0721 (13)	0.0404 (10)	0.0540 (11)	−0.0021 (9)	0.0107 (10)	−0.0030 (8)
C11	0.0552 (11)	0.0587 (13)	0.0703 (14)	0.0132 (10)	0.0037 (10)	−0.0008 (10)
C12	0.0486 (10)	0.0484 (11)	0.0578 (12)	0.0031 (8)	0.0059 (8)	−0.0018 (9)
C16	0.0605 (12)	0.0451 (11)	0.0738 (14)	−0.0038 (9)	−0.0096 (10)	0.0002 (10)
C18	0.0726 (13)	0.0731 (14)	0.0438 (11)	0.0105 (11)	0.0073 (9)	0.0028 (10)
C21	0.0641 (12)	0.0514 (11)	0.0597 (12)	0.0096 (10)	0.0108 (10)	−0.0051 (9)
C20	0.0629 (12)	0.0432 (11)	0.0742 (14)	0.0053 (9)	−0.0028 (10)	0.0043 (10)
C15	0.0634 (13)	0.0569 (13)	0.0885 (17)	−0.0110 (10)	0.0021 (12)	0.0077 (12)
C14	0.0556 (12)	0.0630 (13)	0.0909 (17)	−0.0118 (10)	0.0195 (11)	0.0038 (12)
C19	0.0862 (16)	0.0659 (14)	0.0603 (14)	0.0146 (12)	−0.0002 (12)	0.0210 (11)

### Geometric parameters (Å, °)

**Table:**

C4—C5	1.383 (2)	C10—C9	1.355 (3)
C4—C13	1.429 (2)	C10—C11	1.400 (3)
C4—C3	1.504 (2)	C10—H10A	0.9300
N1—C5	1.380 (2)	C22—C21	1.372 (3)
N1—C1	1.461 (2)	C22—H22A	0.9300
N1—H1	0.850	C9—H9A	0.9300
O1—C3	1.434 (2)	C11—C12	1.364 (3)
O1—C14	1.438 (2)	C11—H11A	0.9300
C13—C12	1.413 (2)	C12—H12A	0.9300
C13—C8	1.423 (2)	C16—C15	1.492 (3)
C5—C6	1.418 (3)	C16—H16A	0.9700
C3—C2	1.526 (2)	C16—H16B	0.9700
C3—H3A	0.9800	C18—C19	1.385 (3)
C8—C9	1.414 (3)	C18—H18A	0.9300
C8—C7	1.414 (3)	C21—C20	1.367 (3)
C6—C7	1.351 (3)	C21—H21A	0.9300
C6—H6A	0.9300	C20—C19	1.355 (3)
C1—C17	1.508 (2)	C20—H20A	0.9300
C1—C2	1.529 (3)	C15—C14	1.494 (3)
C1—H1A	0.9800	C15—H15A	0.9700
C2—C16	1.526 (3)	C15—H15B	0.9700
C2—H2A	0.9800	C14—H14A	0.9700
C7—H7A	0.9300	C14—H14B	0.9700
C17—C18	1.378 (3)	C19—H19A	0.9300
C17—C22	1.387 (2)		
			
C5—C4—C13	119.71 (15)	C9—C10—H10A	120.1
C5—C4—C3	119.05 (15)	C11—C10—H10A	120.1
C13—C4—C3	121.22 (15)	C21—C22—C17	121.24 (18)
C5—N1—C1	120.68 (14)	C21—C22—H22A	119.4
C5—N1—H1	115.1	C17—C22—H22A	119.4
C1—N1—H1	115.7	C10—C9—C8	120.97 (19)
C3—O1—C14	111.34 (15)	C10—C9—H9A	119.5
C12—C13—C8	117.18 (16)	C8—C9—H9A	119.5
C12—C13—C4	122.99 (16)	C12—C11—C10	120.76 (19)
C8—C13—C4	119.82 (15)	C12—C11—H11A	119.6
N1—C5—C4	122.34 (16)	C10—C11—H11A	119.6
N1—C5—C6	118.03 (15)	C11—C12—C13	121.52 (19)
C4—C5—C6	119.60 (16)	C11—C12—H12A	119.2
O1—C3—C4	109.05 (13)	C13—C12—H12A	119.2
O1—C3—C2	110.88 (14)	C15—C16—C2	112.03 (17)
C4—C3—C2	112.49 (14)	C15—C16—H16A	109.2
O1—C3—H3A	108.1	C2—C16—H16A	109.2
C4—C3—H3A	108.1	C15—C16—H16B	109.2
C2—C3—H3A	108.1	C2—C16—H16B	109.2
C9—C8—C7	122.01 (17)	H16A—C16—H16B	107.9
C9—C8—C13	119.76 (17)	C17—C18—C19	120.90 (19)
C7—C8—C13	118.23 (16)	C17—C18—H18A	119.6
C7—C6—C5	121.22 (17)	C19—C18—H18A	119.6
C7—C6—H6A	119.4	C20—C21—C22	120.45 (19)
C5—C6—H6A	119.4	C20—C21—H21A	119.8
N1—C1—C17	109.61 (14)	C22—C21—H21A	119.8
N1—C1—C2	107.90 (15)	C19—C20—C21	119.34 (19)
C17—C1—C2	113.22 (14)	C19—C20—H20A	120.3
N1—C1—H1A	108.7	C21—C20—H20A	120.3
C17—C1—H1A	108.7	C16—C15—C14	109.77 (19)
C2—C1—H1A	108.7	C16—C15—H15A	109.7
C16—C2—C3	109.97 (15)	C14—C15—H15A	109.7
C16—C2—C1	114.33 (16)	C16—C15—H15B	109.7
C3—C2—C1	109.69 (14)	C14—C15—H15B	109.7
C16—C2—H2A	107.5	H15A—C15—H15B	108.2
C3—C2—H2A	107.5	O1—C14—C15	111.70 (17)
C1—C2—H2A	107.5	O1—C14—H14A	109.3
C6—C7—C8	121.39 (17)	C15—C14—H14A	109.3
C6—C7—H7A	119.3	O1—C14—H14B	109.3
C8—C7—H7A	119.3	C15—C14—H14B	109.3
C18—C17—C22	117.36 (17)	H14A—C14—H14B	107.9
C18—C17—C1	120.95 (17)	C20—C19—C18	120.71 (19)
C22—C17—C1	121.68 (16)	C20—C19—H19A	119.6
C9—C10—C11	119.80 (19)	C18—C19—H19A	119.6
			
C5—C4—C13—C12	−177.36 (16)	N1—C1—C2—C3	−59.10 (19)
C3—C4—C13—C12	4.4 (3)	C17—C1—C2—C3	179.41 (14)
C5—C4—C13—C8	1.5 (2)	C5—C6—C7—C8	0.5 (3)
C3—C4—C13—C8	−176.73 (15)	C9—C8—C7—C6	178.50 (18)
C1—N1—C5—C4	−10.1 (3)	C13—C8—C7—C6	−1.1 (3)
C1—N1—C5—C6	171.94 (17)	N1—C1—C17—C18	124.28 (19)
C13—C4—C5—N1	179.96 (16)	C2—C1—C17—C18	−115.2 (2)
C3—C4—C5—N1	−1.7 (3)	N1—C1—C17—C22	−56.2 (2)
C13—C4—C5—C6	−2.2 (3)	C2—C1—C17—C22	64.3 (2)
C3—C4—C5—C6	176.16 (16)	C18—C17—C22—C21	0.8 (3)
C14—O1—C3—C4	175.92 (15)	C1—C17—C22—C21	−178.78 (17)
C14—O1—C3—C2	−59.69 (19)	C11—C10—C9—C8	0.0 (3)
C5—C4—C3—O1	104.13 (17)	C7—C8—C9—C10	−179.42 (18)
C13—C4—C3—O1	−77.58 (19)	C13—C8—C9—C10	0.2 (3)
C5—C4—C3—C2	−19.3 (2)	C9—C10—C11—C12	0.3 (3)
C13—C4—C3—C2	158.98 (15)	C10—C11—C12—C13	−0.7 (3)
C12—C13—C8—C9	−0.6 (2)	C8—C13—C12—C11	0.8 (3)
C4—C13—C8—C9	−179.55 (15)	C4—C13—C12—C11	179.77 (17)
C12—C13—C8—C7	179.06 (16)	C3—C2—C16—C15	−51.1 (2)
C4—C13—C8—C7	0.1 (2)	C1—C2—C16—C15	72.8 (2)
N1—C5—C6—C7	179.13 (18)	C22—C17—C18—C19	−0.2 (3)
C4—C5—C6—C7	1.2 (3)	C1—C17—C18—C19	179.35 (19)
C5—N1—C1—C17	164.33 (16)	C17—C22—C21—C20	−0.9 (3)
C5—N1—C1—C2	40.6 (2)	C22—C21—C20—C19	0.5 (3)
O1—C3—C2—C16	53.87 (19)	C2—C16—C15—C14	52.5 (2)
C4—C3—C2—C16	176.28 (15)	C3—O1—C14—C15	61.8 (2)
O1—C3—C2—C1	−72.68 (18)	C16—C15—C14—O1	−57.3 (3)
C4—C3—C2—C1	49.7 (2)	C21—C20—C19—C18	0.1 (3)
N1—C1—C2—C16	176.86 (15)	C17—C18—C19—C20	−0.2 (4)
C17—C1—C2—C16	55.4 (2)		
